# Unusual recurrence of breast cancer in a BRCA‐variant patient after fat grafting

**DOI:** 10.1002/ccr3.1861

**Published:** 2018-10-31

**Authors:** John P. Skendelas, Clara Lee, Ann Mangino, William E. Carson

**Affiliations:** ^1^ Department of Surgery The Wexner Medical Center at The Ohio State University Columbus Ohio; ^2^ Department of Plastic Surgery The Wexner Medical Center at The Ohio State University Columbus Ohio; ^3^ Division of Surgical Oncology The Wexner Medical Center at The Ohio State University Columbus Ohio

**Keywords:** carcinoma, ductal, reconstruction, recurrence, surgery

## Abstract

The oncologic safety of fat grafting procedures remains well‐characterized in the recent literature; however, we recommend exercising vigilance when evaluating BRCA‐positive and other patients at higher oncologic risk after reconstruction and fat grafting, whose cancer recurrence diagnosis may pose significant clinical challenges.

## 1 INTRODUCTION

Here, we present the case of a patient with T37K BRCA‐variant mutation and unusual pattern of breast cancer recurrence following mastectomy, reconstruction, and early fat grafting. This case highlights the complexity of assessing risk and diagnosis of cancer recurrence in patients with high oncologic risk in the early postoperative period.

Fat grafting (synonyms: adipose tissue grafting, autologous fat grafting, lipofilling, lipotransfer, fat transfer) has emerged as a successful adjunct for breast reconstruction following mastectomy in contemporary plastic surgery practice and is currently supported in the most recent guidelines from the American Society of Plastic Surgeons (ASPS). As of 2015, the ASPS cited Grade B evidence in support of fat grafting for maximizing esthetic outcomes of postmastectomy reconstruction, without increased risk of procedural or long‐term complications including locoregional breast cancer recurrence.^1^ In the short term, patients undergoing fat grafting after mastectomy are at risk of developing fat necrosis and other minor complications. In one study, 23% of patients developed a palpable breast mass after fat grafting at a median time of 10 months postmastectomy with 6% of these cases eventually requiring biopsy.^2^ After cosmetic procedures, clinical providers are placed in the challenging position of determining whether a new breast mass represents a surgical complication or breast cancer recurrence. Several calculators have been developed to aid clinicians in assessing pre‐diagnosis breast cancer risk. These include the Gail, Tyrer‐Cuzick, Claus, and BRCAPRO risk models. However, assessing postmastectomy locoregional recurrence risk in the setting of antiestrogen therapy, chemotherapeutic treatments, and surgical interventions remains challenging and no diagnostic risk calculators are available to aid the clinician. Furthermore, the importance of BRCA‐variant mutations and other genetic abnormalities in modulating the risk of local recurrence have yet to be fully delineated. Breast imaging and invasive biopsies, while necessary for the diagnosis of concerning physical findings, may confer undue stress and morbidity. Conversely, any delay in the diagnosis of local cancer recurrence can be devastating. These parameters require providers to be aggressive yet thoughtful in their assessment of possible recurrences. Here, we present a case of a 33‐year‐old BRCA1‐variant African‐American woman who underwent bilateral mastectomy for clinical stage IIA invasive ductal carcinoma of the right breast with immediate tissue flap reconstruction who developed an unusual pattern of locoregional breast cancer recurrence following fat grafting.

## 2 CASE PRESENTATION

**Figure 1:**
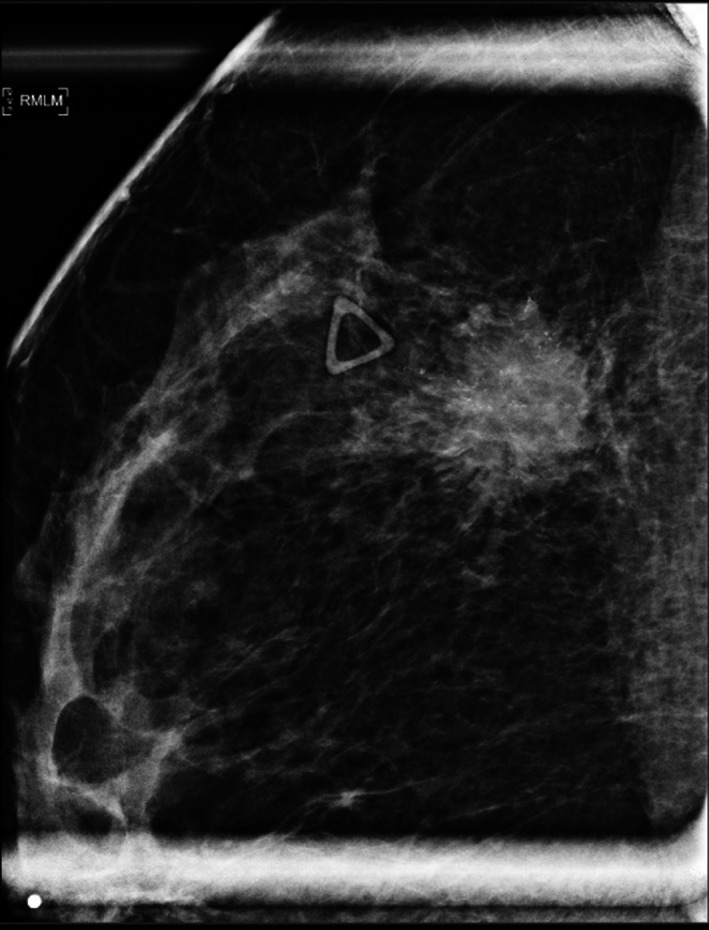
Right mediolateral mammogram (RMLM) of the breast demonstrating a spiculated mass with pleomorphic calcifications in the 10:00 position abutting the pectoralis muscle, BI‐RADS 5

A 33‐year‐old African‐American woman presented to her primary care physician for evaluation of a palpable right breast mass in the upper outer quadrant that she had identified several days prior to presentation. Bilateral mammography demonstrated a spiculated mass with pleomorphic calcifications in the axillary tail of the right breast at the 10:00 position, which corresponded with a palpable 4 cm mass on physical examination (Figure 1). A second, less distinct mass in the upper inner left breast was also visualized. An ultrasound of the right breast demonstrated an irregular hypoechoic and vascular mass measuring 3.3 × 2.1 × 1.9 cm in diameter that abutted the underlying pectoralis major muscle at the 10:00 position in zone 3, 13 cm from the nipple. No abnormalities were identified in the upper inner left breast on subsequent imaging. Taken together, these findings were assigned a BI‐RADS 5 classification. The patient was referred to the breast surgery team at the Stefanie Spielman Comprehensive Breast Center at The Ohio State University Wexner Medical Center for further management.

On establishment of care, the patient revealed a family history of breast cancer involving her maternal cousin, who was diagnosed with left breast cancer at age 27 and right breast cancer at 33 years of age. Of note, her cousin was positive for the T37K variant of uncertain significance in BRCA1, an allele suspicious for deleterious effects. The family history was also significant for a maternal aunt diagnosed with ovarian cancer at 58 years, and a maternal grandmother who developed breast and ovarian cancer during the 7th and 8th decades of life, respectively. On physical examination, the patient had a palpable, firm 3‐4 cm mass within the upper outer quadrant of the right breast. No other masses or nodules were identified in the left or right breast. The nipples appeared normal bilaterally. There was no evidence of cervical, supraclavicular, or axillary lymphadenopathy. These findings were consistent with clinical stage IIA disease. An ultrasound‐guided right core needle biopsy of the dominant breast mass demonstrated a grade 3 invasive ductal carcinoma that was ER‐positive (85%) and PR‐positive (70%). Tumor cells were negative for HER2/neu expression.

After consultation with the breast cancer surgery, medical oncology, and plastic surgery teams, the patient underwent total right breast mastectomy for invasive ductal carcinoma with sentinel lymph node biopsy and prophylactic total left breast mastectomy followed by immediate reconstruction. Given the patient's choice of procedure, it was felt that a referral to medical genetics could be made postoperatively. Two right axillary sentinel lymph nodes were identified via nuclear scintigraphy, but these were negative for carcinoma on intraoperative consultation with pathology. Following resection, the patient underwent immediate bilateral breast reconstruction with muscle‐sparing free transverse rectus abdominis myocutaneous (MS‐TRAM) flaps and implantation of prosthetic mesh in the abdomen without complication. An attempt was made to perform a reconstruction based on a superficial inferior epigastric artery flap (SIEA), but no usable segment of the SIEV was identified. The patient tolerated the procedure well, and the remaining hospital course was unremarkable. Pathologic evaluation of the right breast revealed a grade 3 invasive ductal carcinoma measuring 2.7 cm in diameter along with high nuclear grade ductal cell carcinoma in situ. Margins were clear on final pathology with a 1.1 cm margin. Evaluation of the left breast demonstrated benign parenchyma with usual type ductal hyperplasia, apocrine metaplasia, and cyst formation without evidence of carcinoma.

Due to her African‐American ancestry, early age of presentation and strong family history of breast and ovarian malignancy the patient were referred to clinical cancer genetics for counseling. Testing revealed that the patient was indeed positive for the T37K variant in the BRCA1 gene. This was considered a deleterious mutation in the setting of known malignancy and strong family history of breast and ovarian cancer. The patient was definitively diagnosed with hereditary breast and ovarian cancer syndrome, and it was recommended she begin ovarian suppression and endocrine therapy. At this time, the patient's Oncotype Dx score was 18, indicative of a 12% likelihood of breast cancer recurrence within 10 years of initial diagnosis. Two months after mastectomy, the patient underwent placement of a PR ParaGard Copper IUD for ovarian suppression and started tamoxifen 20 mg daily. Prophylactic bilateral oophorectomy was also offered, but the patient declined at this time. The patient cited an interest in deferring this surgery until after her childbearing years. She declined adjuvant systemic chemotherapy as well, having been offered a regimen containing doxorubicin and cyclophosphamide.

The patient underwent further breast reconstruction 3.5 months postmastectomy that included bilateral nipple reconstruction and fat grafting. The patient was found to have a contour irregularity in the superolateral aspects of the bilateral‐free flaps (left greater than right) at the junction of the mastectomy skin and the edge of the flap. Using standard Coleman technique, 30 cc of lidocaine with epinephrine was injected into the lateral aspect of the abdominal donor site. After allowing sufficient time for vasoconstrictive effect, approximately 68 cc of processed fat was harvested and processed by rolling the harvested fat on a Telfa pad. This material was then injected into the superior pole concavity at multiple sites bilaterally. The abdominal donor site was revised without complications. The patient presented to plastic surgery clinic 6.5 months after mastectomy for evaluation of two palpable nodules in the upper outer quadrant of the right reconstructed breast at the 9 and 10:00 positions. The nodules were thought to be consistent with fat necrosis in the postsurgical setting, and no further workup was obtained.

**Figure 2:**
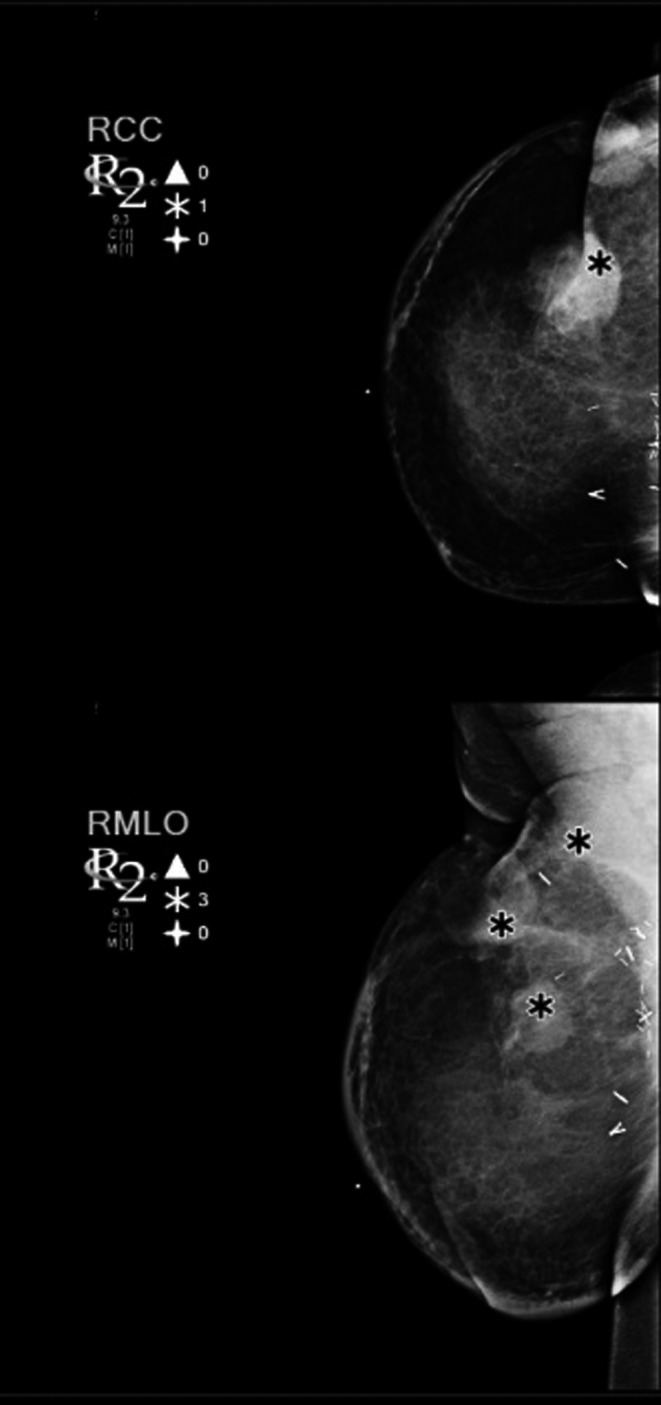
Cranial‐caudal (CC) and mediolateral oblique (MLO) views of the right breast are shown demonstrating four discrete breast masses (*), BI‐RADS 4

**Figure 3:**
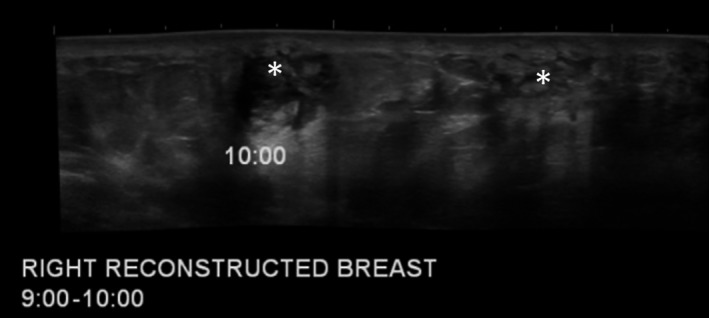
Representative ultrasonographic evaluation at the time of digital mammogram of the Z3 region of the right reconstructed breast (Figure 2) demonstrating mixed echogenic masses (*) at the 9‐10:00 position

**Figure 4:**
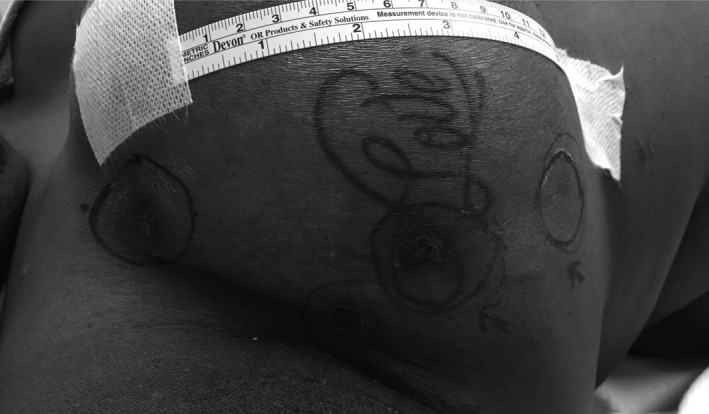
Preoperative image of the right reconstructed breast demonstrating three discrete superficial lesions corresponding to known locoregional cancer recurrence

Four months later (7.5 months postmastectomy), the patient underwent repeat fat grafting to correct bilateral breast contour irregularities. For this procedure, the hips were chosen as the fat graft donor site and the Coleman technique was again utilized. In total, 61 cc of fat was injected into contour deformities of the upper and lateral aspects of the reconstructed left breast, and 27 g of fat was injected into similar irregularities of the right breast without complication. The patient recovered well and was pleased with the cosmetic outcome. The patient presented for follow‐up of 5 months following the second fat grafting procedure (12 months postmastectomy) with four nodules at the periphery of the right reconstructed breast that were located at evenly spaced intervals from the 9:00 to 12:00 axes. These areas were believed to be fat necrosis. Again, no further workup was obtained. Approximately fifteen months postmastectomy, the patient was evaluated for rising CA‐125 levels by her OB‐GYN physician due to concern for possible BRCA‐associated ovarian cancer. The initial CA‐125 level was 12 at 2.5 months postmastectomy and increased to 81 by 15 months postmastectomy. Workup for the progressive increase in CA‐125 of unknown etiology included transvaginal ultrasound and CT scan of the abdomen and pelvis. These scans were negative for gynecological malignancy but demonstrated significant skin thickening of the right reconstructed breast with nodularity involving the lateral aspect of the underlying muscle. Follow‐up mammogram (Figure 2) and ultrasound (Figure 3) of the reconstructed breast demonstrated several suspicious masses. These included a mixed echogenic mass measuring 2.8 × 2.0 × 2.6 cm at the 9:00 position, a mixed echogenic mass measuring 3.0 × 1.7 × 2.8 cm at the 9‐10:00 position, a hypoechoic mass measuring 1.7 × 1.4 × 2 cm at the 10:00 position, and a fourth mass measuring 3.4 × 2.8 × 4 cm at the 12:00 position of the right reconstructed breast. The mammogram received a BI‐RADS 4 classification. These masses were located at equal intervals in a symmetrical arc in the extreme upper outer quadrant of the right breast (see Figure 4).

The patient underwent ultrasound‐guided biopsy of the suspicious masses in the right breast at the 9:00, 9‐10:00, and 12:00 positions of zone 3. In total, 3 of the 4 masses were biopsied. The pathologic diagnosis of each specimen was consistent with grade 3 invasive ductal carcinoma. Estrogen receptors and progesterone receptors were positive at 70% and 60%, respectively. HER2/neu expression was negative. Staging CT scans of the chest demonstrated a dominant centrally necrotic mass with peripheral rim enhancement in the superior aspect of the right reconstructed breast and multiple other right subcutaneous nodules. Multiple enlarged right axillary and subpectoral lymph nodes were also observed. A whole body bone scan was negative for distant metastatic bone disease, with the exception of increased uptake noted in the L4 vertebral body. MRI evaluation of the lumbar spine was negative for contrast‐enhancing lesions. The locoregional recurrence was consistent with clinical stage IIIC disease (T4a, pN3b, M0).

The patient was referred to medical oncology, and given the extent of disease; it was felt that pre‐operative chemotherapy would assist the surgeon in obtaining margins when the area of recurrence was resected. The patient's systemic chemotherapy regimen consisted of six cycles of adriamycin/cyclophosphamide and three cycles of dose‐dense paclitaxel. This treatment was initiated 17.5 months postmastectomy. The patient tolerated chemotherapy well without significant side effects. Interval imaging after 2.5 months of treatment demonstrated a decrease in the size of the four lesions to 1.3 × 1.0 × 1.5 (from 2.8 × 2.0 × 2.6 cm) at the 9:00 position, 1.6 × 0.9 × 1.3 (from 3.0 × 1.7 × 2.8 cm) at the 9‐10:00 position, 1.5 × 1.5 × 1.9 (from 1.7 × 1.4 × 2 cm) at the 10:00 position, and 1.5 × 1.6 × 2.1 (from 3.4 × 2.8 × 4 cm) at the 12:00 position of the right reconstructed breast. En bloc resection of the right chest wall recurrences and right axillary lymph node dissection were performed approximately 1 month after the completion of chemotherapy. The recurrent cancers were encompassed within a single oval‐shaped portion of chest wall, and the axillary contents were resected in continuity with this tissue.

The final pathology on the resection specimen revealed the presence of residual invasive ductal carcinoma, indicating an incomplete response to chemotherapy. Invasive carcinoma was present as clusters of individual tumor cells spread over three areas with maximum dimensions of 2.5, 1.8, and 1.4 cm. It was felt as this time that two of the previously identified masses at the 10:00 axis had become confluent during the course of chemotherapy. Therapy‐related changes and biopsy site changes were seen, and all margins were negative for carcinoma. The invasive carcinoma was 0.5 cm from the closest superior margin. Macrometastatic carcinoma was identified in four lymph nodes. The largest tumor deposit measured 0.5 cm in greatest dimension and exhibited extracapsular extension. Four additional lymph nodes with isolated tumor cells and thirteen lymph nodes negative for carcinoma were also present. The invasive tumor was HER2‐negative (IHC score: 0) and estrogen receptor and progesterone receptor‐positive. The patient then underwent 6 weeks of daily radiation therapy to the right chest wall without any complications. Following completion of radiotherapy, the patient resumed chemotherapy that consisted of exemestane and goserelin for aromatase inhibition and ovarian suppression, respectively. The patient continues to follow‐up with the multidisciplinary breast cancer team.

## 3 REVIEW OF THE LITERATURE AND DISCUSSION

The present report summarizes the case of a BRCA1‐variant patient with stage IIA invasive ER+/PR+ductal carcinoma who developed locoregional recurrence after mastectomy, breast reconstruction, and fat grafting. These masses were initially thought to be fat necrosis. Due to persistence and interval increase in the number of the breast nodules several months following her initial surgery, the patient underwent biopsies of three of the four sites of concern and these results showed the presence of hormone receptor‐positive invasive ductal carcinoma.

The unfortunate early recurrence of this patient's breast cancer highlights the difficulty in the evaluation of breast masses postmastectomy, and in this particular setting, after immediate and follow‐up breast reconstruction. Fat necrosis is a well‐documented phenomenon that occurs following breast trauma and surgical procedures, affecting 4.4% of patients at a median follow‐up of 18 months,^3^ and 6.9% of patients with myocutaneous flaps in another report.^4^ In one study, 23% of patients developed a palpable breast mass after fat grafting at a median time of 10 months postmastectomy with 6% of these cases eventually requiring biopsy.^2^ Clinically, palpable foci of fat necrosis are difficult to diagnose accurately in the absence of radiologic evaluation or biopsy. To address this concern, several classification schemes have recently been developed to differentiate benign from malignant breast lesions in postmastectomy patients with suspected fat necrosis using ultrasound.^5^, ^6^

Although this patient developed breast masses that were evident on physical examination prior to follow‐up imaging, many patients undergo oncological surveillance via radiologic evaluation. A recent retrospective study demonstrated that the prevalence of breast imaging (including mammography, ultrasound, mammography plus ultrasound, MRI) was 31.7% in postmastectomy patients with a mean follow‐up of 20.2 months after fat grafting. The indications were routine oncological evaluation following reconstruction (53% of imaged breasts), palpable breast mass (26%), and persistent breast pain (9%). Of these examinations, 41.5% of breast imaging findings were unremarkable, 43.4% were consistent with benign findings including fat necrosis, and 7.5% were suspicious and prompted biopsy. In that series, all biopsies were negative for malignancy and the overall rate of biopsy was 4.8%.^7^ Due to the frequency of suspicious breast findings, several algorithms have been proposed in an attempt to standardize the management of postmastectomy chest wall masses.^2^ After a review of the literature, these studies highlight the efficacy of radiologic evaluation in differentiating benign from malignant breast lesions and an acceptably low biopsy rate if imaging findings are suspicious or equivocal.

While the possibility of malignancy in any patient with a history of breast cancer presenting with new masses should be considered, it is unusual for a patient with resected breast cancer to present with locoregional recurrence 15 months after definitive surgery. Even more unusual, the patient initially presented with two palpable breast masses 6.5 months postmastectomy (3 months following the first fat grafting procedure) prior to undergoing the second fat grafting procedure approximately one month later. Before biopsy, there was a latent period of 8 months in which the patient's CA‐125 level increased from 33 to 81; therefore, her breast cancer recurrence may have occurred as early as 6.5 months postmastectomy. While the patient declined systemic chemotherapy, there is no definitive evidence to suggest that this decision increased her risk of recurrence. Her Oncotype Dx score was 18, indicative of a 12% risk of recurrence within 10 years. For comparison, the overall risk of locoregional recurrence for patients undergoing mastectomy is 9% over 10 years^8^; and in cases of local recurrence, malignancy usually develops a median of 2‐3 years postmastectomy^9^ with 75% of cases developing within 5 years.

This patient represents a difficult case with respect to clinical management as there are no National Comprehensive Cancer Network (NCCN) guidelines for cancer surveillance of the reconstructed breast following mastectomy. As discussed previously, fat necrosis is a common complication after mastectomy, tissue flap reconstruction, and fat grafting. Also, the patient had several general risk factors for the development of fat necrosis—obesity and previous abdominal surgery (history of cholecystectomy).^4^ This discussion is particularly relevant due to the fact that the four sites of breast mass formation occurred near or at the sites that received early fat grafting during reconstruction.

After years of debate, there is a wealth of evidence demonstrating that fat grafting confers no increased oncological risk in larger studies.^10^ The aim of this report is not to call these studies into question. Rather, this report highlights several factors which complicated the diagnosis of this patient's unusual pattern of recurrence. From our review of the literature, the median time from cancer surgery to fat grafting is highly variable and often not reported. In the cases where this information is available, the time may vary from a median 16.7 months^2^ to 36 months,^11^ and up to 48 months^12^ following primary cancer resection. Our patient received her first fat graft 3.5 months postmastectomy, well before the 2‐3 year median time of local breast cancer recurrence.^9^ This is somewhat uncommon timing compared to the literature. As previously discussed, the overall risk of locoregional recurrence for patients undergoing mastectomy is 5%‐10% over 10 years; however, for BRCA‐positive patients, this risk increases to 15% and 25% after 5 and 10 years, respectively.^13^ For the patient discussed in this report, the BRCA‐associated risk of locoregional recurrence is more difficult to assess. As previously noted, the patient underwent genetic testing and was determined to have a T37K variant, a missense mutation in the BRCA1 ring finger domain. In this patient, the T37K variant met the classification criteria for a deleterious mutation.^14^ However, it is unknown whether the T37K variant for this patient would confer the same breast cancer recurrence risk profile as BRCA nonsense mutations.

## 4 CONCLUSION

Here, we have presented the case of a 33‐year‐old BRCA1‐positive African‐American woman who developed locoregional recurrence of invasive ductal carcinoma at 15 months postmastectomy but whose clinical presentation may have been consistent with recurrence as early as 6.5 months postmastectomy. These four recurrent breast masses were significant for appearing to mirror the location of fat grafting. It is also important to note that these palpable masses were evident after one fat grafting procedure. The clinical course of this patient underscores the importance of a careful evaluation of all breast masses in breast cancer patients postmastectomy and reconstruction. The purpose of this report is to highlight an unusual pattern of breast cancer recurrence in a BRCA‐variant patient with high‐risk factors for recurrence. The diagnosis was complicated by the anticipated side effects of fat grafting which is a well‐established reconstructive procedure. Like the authors whose studies are cited in this report, we believe that the appropriateness and timing of fat grafting should be decided upon in a multidisciplinary fashion by the surgical oncology, plastic surgery, and medical oncology teams on a case‐by‐case basis. Since BRCA‐positive and variant patients are often younger patients who may seek breast reconstructive options for esthetic purposes, we believe that the possibility of breast cancer recurrence should always remain at the forefront of the differential diagnosis in the case of an unusual clinical presentation.

## AUTHOR CONTRIBUTION

JPS: performed the patient chart review, literature review, and wrote the manuscript under the guidance and recommendations of the following coauthors and principal investigator. CL: provided expert consultation in regard to understanding the role of reconstruction and fat grafting following breast surgery. AM: provided the patient consent information and administrative support in retrieving additional patient information for use in the clinical history that was otherwise unavailable in the accessible electronic medical record. WEC: developed the original concept for the manuscript and provided the most significant contributions regarding revisions and critical review of the manuscript. All of the authors were provided manuscript drafts throughout the writing process in order to aid the development of the final submission.

## CONSENT

Informed consent was obtained prior to the publication of this case report.
